# Next-Generation Heterocyclic Electrophiles as Small-Molecule Covalent MurA Inhibitors

**DOI:** 10.3390/ph15121484

**Published:** 2022-11-29

**Authors:** Péter Ábrányi-Balogh, Aaron Keeley, György G. Ferenczy, László Petri, Tímea Imre, Katarina Grabrijan, Martina Hrast, Damijan Knez, Janez Ilaš, Stanislav Gobec, György M. Keserű

**Affiliations:** 1 Medicinal Chemistry Research Group, Research Centre for Natural Sciences, Magyar tudósok krt 2, H-1117 Budapest, Hungary; 2 MS Metabolomics Research Group, Research Centre for Natural Sciences, Magyar tudósok krt 2, H-1117 Budapest, Hungary; 3 Faculty of Pharmacy, University of Ljubljana, Askerceva 7, SI-1000 Ljubljana, Slovenia

**Keywords:** covalent inhibitor, heterocyclic electrophiles, cysteine labelling, quaternization, MurA

## Abstract

Heterocyclic electrophiles as small covalent fragments showed promising inhibitory activity on the antibacterial target MurA (UDP-N-acetylglucosamine 1-carboxyvinyltransferase, EC:2.5.1.7). Here, we report the second generation of heterocyclic electrophiles: the quaternized analogue of the heterocyclic covalent fragment library with improved reactivity and MurA inhibitory potency. Quantum chemical reaction barrier calculations, GSH (*L*-glutathione) reactivity assay, and thrombin counter screen were also used to demonstrate and explain the improved reactivity and selectivity of the *N*-methylated heterocycles and to compare the two generations of heterocyclic electrophiles.

## 1. Introduction

Heterocycles are part of the main structural elements of bioactive compounds due to their ability to interact with the targeted protein [1,2]. These cores can be equipped with electrophilic warheads mostly targeting cysteine, and thus the use of heterocyclic electrophiles as targeted covalent inhibitors (TCIs) and covalent fragments emerged in recent years [3,4,5,6,7,8,9,10,11,12,13]. In particular, we have shown that warheads with low reactivity can be activated by the electron-withdrawing character of the heterocycle [14,15,16]. In fact, *N*-methylation of 4-bromo or 2-vinylpyridine resulted in even higher thiol-reactivity due to the increased electron-withdrawing character of the positively charged heteroatom, and quaternized heterocyclic electrophiles could be used for protein labelling [17,18]. These results prompted us to rationally assemble a library of methylated electrophilic heterocycles that could provide suitable fragment starting points for covalent drug discovery programs.

Usually, the used electrophilic warhead contains at least three (e.g., isothiocyanate), and in most cases five-to-nine (e.g., seven in the case of acrylamide) atoms [7,19,20]. Thus, equipping a non-covalent hit with a warhead of several atoms could significantly influence the shape and the binding mode. On the contrary, we have already shown that using a warhead with only one or two atoms (e.g., halogens, nitrile, vinyl, acetylene) may affect the binding mode less [14,15]. As the selectivity of the ligand is majorly influenced by the interactions of the non-covalent scaffold, promiscuity is even more avoided by staying close to the recognized structural elements. However, these functional groups (i.e., halogens, nitrile, vinyl, acetylene) on an aromatic core are usually not reactive enough for aromatic nucleophilic substitution or a nucleophilic addition, but an electron-withdrawing heterocyclic core can be applied to reach sufficient reactivity. We have shown that by assembling a rationally designed library of heterocyclic electrophiles using several five- and six-membered heterocycles and the aforementioned warheads, a wide reactivity of the covalent fragments can be covered, which can be tuned by the careful choice of the heterocycle–warhead combination [14,15,21]. On the basis of our former fragment screening on MurA (UDP-N-acetylglucosamine 1-carboxyvinyltransferase, EC:2.5.1.7), which resulted in several hits, but only one with single-digit micromolar activity, we decided to apply the second generation of the electrophilic heterocycles obtained by systematic methylation of the library members. Commercially available five- or six-membered heterocycles equipped with six different warheads (Cl, Br, I, nitrile, vinyl, acetylene) were selected. We investigated the efficacy of this library on MurA, tested the GSH (*L*-glutathione) reactivity of the identified hits, and explained the difference in reactivity between the two generation of the library by quantum chemical calculations of the activation barrier of their reaction with methyl thiolate. This approach led us to new single digit micromolar MurA inhibitor fragments that could be considered as viable starting points for novel MurA inhibitor chemotypes. These results and the characterized library reported here could initiate further studies in this direction.

## 2. Results

**Scheme 1:**
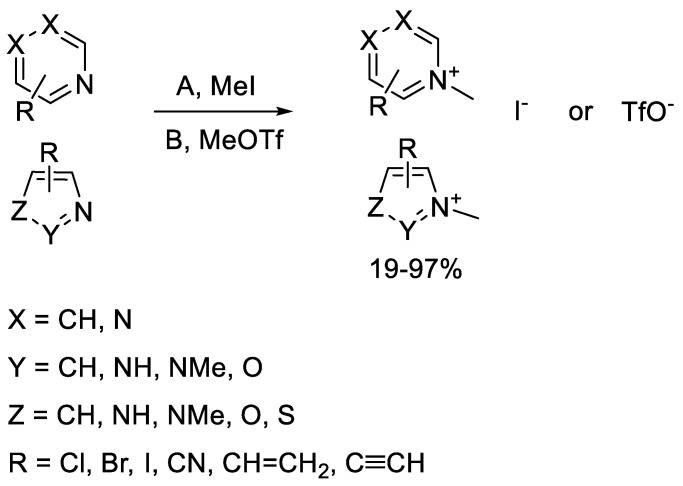
General scheme for the methylation of the heterocyclic electrophiles.

The second generation of our library of methylated electrophilic heterocycles contained pyridines, pyrimidines, pyrazines, imidazoles, pyrazoles, oxazoles, thiazoles, and isoxazoles substituted in various positions. The electrophilic moieties were Cl, Br, and I atoms reacting in aromatic nucleophilic substitution, or nitrile, vinyl, and ethynyl groups reacting in nucleophilic addition [14,15]. The methyl group was incorporated using methyl iodide or methyl trifluoromethanesulfonate. In most cases, the reactions went smoothly, resulting in acceptable yields (17–99%) after a simple filtration or evaporation of the solvent. In the case of the imidazoles and pyrazoles, both nitrogen atoms were methylated. The products were iodide or triflate salts, and finally the library contained a total of 57 compounds (Scheme 1).

**Figure 1:**
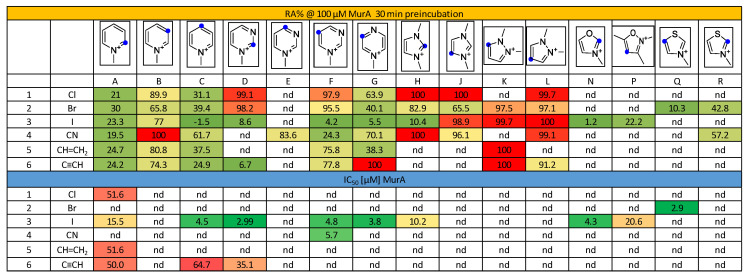
Inhibitory activity of methylated electrophilic heterocycles against MurA represented in residual activity (RA %) after 30 min preincubation and IC_50_ values (μM). Blue dots indicate the position of the warhead. Coloring is in line with the activity, from red (low) through yellow (moderate) to green (high). nd stands for “not determined”.

Investigating the first generation of the library (84 compounds) in a MurA biochemical assay, we identified 23 hits that had less than 70% residual activity (RA) at a screening concentration of 500 μM. Four of the active inhibitors showed IC_50_ < 100 uM against MurA, and only a single hit exhibited IC_50_ < 50 μM (**F3**, 3.8 µM) [11]. The bioactivity of the second generation of the library, consisting of 57 quaternized heterocyclic electrophiles, was tested and compared to the activity of the non-methylated compounds. The quaternized library provided 30 fragments with RA < 70% already at 100 μM concentration, and 15 compounds had IC_50_ < 100 μM (26% hit rate), including 11 fragments with IC_50_ < 50 μM (Figure 1). 

The 2- and 4-pyridinium compounds (Figure 1 columns A and C) were more active than the 3-pyridiniums (Figure 1 column B). Comparison of the IC_50_ data showed that activity increased in parallel with the increasing number of the nitrogen atoms. Among the six-membered heterocycles, the compounds with iodine warhead with single-digit micromolar IC_50_s (**C3+**, **D3+**, **F3+**, **G3+**) were the most active, suggesting that the warhead reactivity in this case of is less dependent on the heterocycle. Among the five-membered heterocycles, the oxazoles and thiazoles (**N3+**, **P3+**, **Q2+**, **R2+**, **R4+**) performed best, mostly with low-micromolar IC_50_ values, and among the imidazoles and pyrazoles, 2-iodoimidazolium (**H3+**) showed considerable activity.

Methylated hits were subjected to an NMR-based single-point GSH-assay, measuring the conversion of heterocycles after 15 min. We found that 12 of the 15 most active fragments were GSH active (showing >50% conversion after 15 min, see Table S1). On the one hand, these results showed that the GSH assay for the methylated library was a good indicator of bioactivity, which could be explained by the high nucleophilicity of the available cysteine residue on MurA. On the other hand, the limited GSH activity of some hits might suggest favorable stability, which is a good starting point for further development. 

Comparison of the non-methylated and methylated compound pairs reveals that the quaternary methylation enhanced the reactivity of most of the active fragments and increased the affinity to the low micromolar range in many cases (Table 1). We could conclude that the second-generation heterocycles exhibited a 5–100-fold increase in most cases; the only exception was **F3**, which was the most active in the previous study (Table 1). We might highlight compounds **C3+** and **Q2+**, where the activity increased to 25-fold. The highest increase (100-fold) was observed by 4-iodopyridinium (**D3+**). To explain the observed biochemical activity, we chose fragment **C3+** and proved the single and double labelling on MurA by intact MS measurements (Figure S1).

Next, we tested the cysteine selectivity of fragment hits **A1+**, **A3+**, **A5+**, **A6+**, **C3+**, **C6+**, **D3+**, **D6+**, **F3+**, **F4+**, **G3+**, and **Q2+** by investigating the biochemical activity against thrombin, a serine protease that contains no cysteine in the active site (Table S1). At 1 mM concentration, only fragments **C3+** and **D6+** showed RA < 50%. These data are in favor of significant cysteine selectivity of the methylated heterocyclic compounds.

**Scheme 2:**
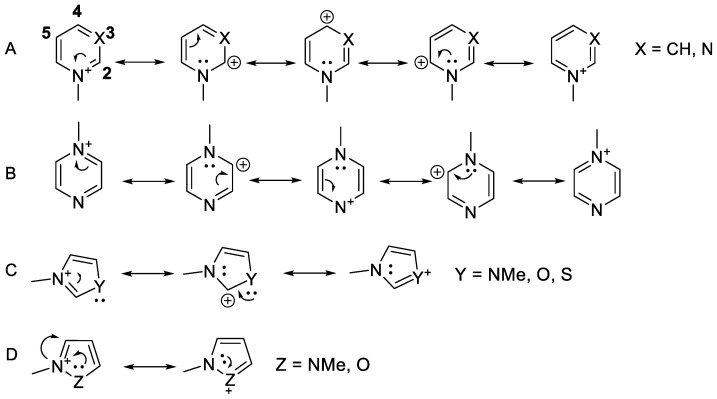
Mesomeric structures of the *N*-methylated heterocycles. (**A**) Pyridiniums (Figure 1 columns A–C) and pyrimidiniums (Figure 1 columns D–F); (**B**) pyraziniums (Figure 1 column G); (**C**) imidazoliums (Figure 1 column H), oxazoliums (Figure 1 column N), and thiazoliums (Figure 1 columns Q and R); (**D**) pyrazoliums (Figure 1 columns K and L) and isoxazolium (Figure 1 column P).

Notably, the observed reactivity pattern against MurA was consistent with the position of the positive charge in the aromatic ring (Scheme 2). The compounds with no presumed positive charge on the warhead-substituted carbon, particularly the 3-pyridiniums (Figure 1 column B), 5-pyrimidiniums (Figure 1 column F), 5-imidazoliums (Figure 1 column J), and 3- and 4-pyrazoliums (Figure 1 columns K,L), were mostly not active.

Next, we aimed to determine the Gibbs free energies of activation for some representative fragment–thiolate reactions. We chose the six-membered heterocycles with Cl warhead (**A1+-G1+**) to compare methylated and non-methylated pairs, and compounds **A1+-A6+**, **F3+** and **F4+** to compare the other warheads (Table 2). The heterocycles were reacted with methyl thiolate anion or cysteamine using the M06-2x/6-31+G** (d,p) [22] or def2SVP basis sets [23] with the implicit solvent effect of water (IEFPCM) [24] considered in the Gaussian09 program package [25]. The activation barriers of the methylated heterocycles were found to be significantly lower than those of the corresponding unmethylated heterocycles, in line with the expected higher reactivity of the *N*-methylated compounds. In particular, the Gibbs free energies of activation (ΔG^#^) decreased significantly with methylation from 68–135 kJ mol^−1^ to 0.3–72 kJ mol^−1^. Comparing the same heterocycles with warheads in different positions, we were able to conclude that among pyridines (**A1-C1**) and pyrimidines (**D1-F1**), the less reactive ones (**B1** and **F1**) had the highest ΔG^#^ in the series. With the increase in the heteroatoms in the ring from one (**A1-C1**) to two (**D1-G1**), the average ΔG^#^ decreased from 56.4 kJ mol^−1^ to 9.8 kJ mol^−1^, which could be explained by the higher activating effect of more nitrogen atoms. However, no transition state for S_N_Ar reactions of the 5-bromo-substituted pyrimidinium (**F2+**) was identified; rather, the thiolate group approached the C6-atom adjacent to the methylated *N*-atom before it moved toward C5 (holding the halogen atom) with steeply increasing energy. By contrast, the reaction between 5-iodopyrimidinium (**F3+**) and MeS^-^ proceeded with no barrier, suggesting that the reactivity of the 5-iodo-derivative is sufficient for cysteine labeling. A small barrier was found for the 5-cyanopyrimidine (**F4+**)-MeS^-^ reaction, while no transition state could be identified for the analogous reaction of 5-vinyl- and 5-ethynyl derivatives (**F5+** and **F6+**, respectively). Barriers were also calculated for the reaction between **A1+** and either MeSH or MeOH. The obtained free energies were 177.6 kJ mol^−1^ and 102.3 kJ/mol, respectively. The largely increased barrier with MeSH compared to that of with MeS^-^ suggests that the deprotonation of cysteine largely facilitated the reaction. The high barrier with MeOH suggests that **A1+** did not react with either serine or threonine residues that are expected to be present in protonated form.

In summary, the calculated barriers of *N*-methylated compounds suggest the ability to react with deprotonated cysteine residues. Note that the barriers were comparable both for the S_N_Ar reactions of halogen-substituted *N*-methylated heterocycles and for the nucleophilic addition on cyano/vinyl/ethynyl-substituted *N*-methylated heterocycles.

## 3. Materials and Methods

### 3.1. General Experimental Procedures

All chemicals and solvents with >95% purity were purchased from commercial vendors (Sigma-Aldrich (Budapest, Hungary), Fluorochem (Hadfield, UK), Combi-Blocks (San Diego, CA, USA)) and used without further purification. ^1^H NMR and ^13^C NMR spectra were recorded in DMSO-d_6_, CD_3_CN, or D_2_O solution at room temperature on a Varian Unity Inova 500 spectrometer (500 and 125 MHz for ^1^H NMR ^13^C NMR spectra, respectively), with the deuterium signal of the solvent as the lock. Chemical shifts (δ) and coupling constants (J) are given in ppm and Hz, respectively. HPLC-MS measurements were performed using a Shimadzu LC-MS-2020 device equipped with a Reprospher-100 C18 (5 µm; 100 × 3 mm) column and a positive–negative double ion source (DUIS±) with a quadrupole MS analyzer in a range of *m*/*z* 50–1000. The sample was eluted with gradient elution using eluent A (0.1% formic acid in water) and eluent B (0.1% formic acid in acetonitrile). Flow rate was set to 1 mL/min. The initial condition was 0% B eluent, followed by a linear gradient to 100% B eluent by 1 min; from 1 to 3.5 min, 100% B eluent was retained, and from 3.5 to 4.5 min, we went back to 5% B eluent, and this was retained from 4.5 to 5 min. The column temperature was kept at room temperature, and the injection volume was 1–10 µL. Purity of compounds was assessed by HPLC with UV detection at 254 nm; all tested compounds were >95% pure. High-resolution mass spectrometric measurements were performed using a Q-TOF Premier mass spectrometer (Milford, MA, USA) in positive or negative electrospray ionization mode. Reactions were monitored with Merck silica gel 60 F^254^ TLC plates (Darmstadt, Germany). The column chromatography purifications were performed using Teledyne ISCO CombiFlash Lumen+ R_f_.

Synthetic procedures together with the corresponding NMR data are available in the Supplementary Materials.

### 3.2. GSH Assay Based on NMR

For the NMR assay, a 500 mM D_2_O stock solution was first prepared for each fragment along with an 800 mM D_2_O stock solution of GSH. Thereafter, a 5 mM electrophilic standard solution was prepared by adding 20 μL of stock solution to a 2 mL 100 mM potassium hydrogen phosphate/sodium hydrogen phosphate buffer solution (pH 7.4). A total of 0.5 mL of this electrophilic standard solution was placed in an NMR tube, and then the ^1^H NMR was recorded. The peak values obtained during the measurement were considered the zero-point measurement or reference peaks of the reactivity study. For the reactivity measurement, 20 μL of the fragment stock solution was added to 1980 μL phosphate buffer solution (pH 7.4) along with 20 μL of GSH stock solution (final concentration = 80 mM GSH and 5 mM fragment). The contents were vortexed until homogenization and were transferred to an NMR tube. The resulting mixture was analyzed by ^1^H NMR measurements after 15 and 30 min, and 4 and 24 h. The AUC (area under the curve) values were determined via integration of the corresponding peaks in the NMR spectra.

Expression and purification of *E. coli* MurA (UDP-N-acetylglucosamine 1-carboxyvinyltransferase, EC:2.5.1.7) was performed according to the known procedure [26]. Briefly, the MurA plasmid was used to transform chemically competent *Escherichia coli* NiCo21(DE3) (New England Biolabs, Ipswich, MA, USA). The transformant was cultured at 37 °C and 250 rpm in LB broth. Expression was induced by addition of 1 mM IPTG and cultured for an additional 3 h. Cells were harvested by centrifugation (3000× *g* for 10 min at 4 °C), and cell pellets were stored at −80 °C until purification. Cell pellets were resuspended in buffer 50 mM Tris (pH 8), 150 mM NaCl, and 10 % glycerol and lysed on ice by sonication. Cell debris was removed by centrifugation for 30 min (16,000× *g*, 4 °C, repeated twice). The lysate was loaded onto a HiTrap 1 mL IMAC HP column (Cytiva, Marlborough, MA, USA). The column was washed with buffer containing 50 mM imidazole with 20 column volumes, and the protein was eluted with buffer containing 250 mM imidazole. The eluted MurA was exchanged into proprietary buffer (50 mM Tris (pH 8), 150 mM NaCl, 10 % glycerol).

### 3.3. MurA Inhibitory Assay

The inhibition of the MurA enzyme was monitored using the colorimetric malachite green method in which orthophosphate generated during the reaction is detected [27]. The assay was performed in 96-well microplates with a final volume of 50 μL. The MurA enzyme was preincubated with the substrate UNAG (UDP-N-acetylglucosamine, Sigma, St. Louis, MO, USA) and compound for 30 min at 37 °C. The reaction was started by the addition of the second substrate PEP, resulting in a mixture containing buffer (50 mM HEPES, pH 7.8), 0.005% Triton X-114, 200 μM UDP-N-acetylglucosamine, 100 μM phosphoenolpyruvate, purified MurA (diluted in 50 mM HEPES, pH 7.8), and the test compounds dissolved in DMSO (5%) at 100 µM. After incubation for 15 min at 37 °C, the enzyme reaction was ended by adding Biomol^®^ reagent (100 µL), and the absorbance was measured at 650 nm after 5 min using a microplate reader (Synergy H4, BioTek Instruments, Inc., Winooski, VT, USA). All of the experiments were run in duplicate. Residual activities (RAs) were calculated with respect to similar assays without the tested compounds and with 5% DMSO. IC_50_ values were determined by measuring residual activities at 7 different compound concentrations and calculated using GraphPad Prism (GraphPad Software, San Diego, CA, USA).

### 3.4. MurA Labelling

The covalent labelling procedure was conducted as described earlier with slight modifications [15]. First, 42 μM stock solution of MurA in 20 mM HEPES at pH 7.2–7.4 with 1 mM DTT (dithiothreitol, Sigma) was filtered through a G25 desalting column, and the medium was changed to 50 mM Tris with 0.005% Triton X-100 at pH 8.0. For the activation of the enzyme, 1 mg UNAG was added as a solid to reach 40 mM concentration, and the mixture was incubated at 37 °C for 30 min. Fragments were added from a 100 mM DMSO stock diluted in the labelling solution to 5 mM. The incubation was continued at 37 °C for an additional 60 min. After the labelling, the mixture was purified on a G25 desalting column.

The molecular weights of the conjugates were identified using a Triple-TOF 5600+ hybrid Q-TOF LC/MS/MS system (Sciex, Singapore, Woodlands) equipped with a Duo Spray Ion Source coupled with a Shimadzu Prominence LC20 UFLC (Shimadzu, Kyoto, Japan) system consisting of a binary pump, an autosampler, and a thermostated column compartment. Data acquisition and processing were performed using Analyst TF software version 1.7.1 (AB Sciex Instruments, Redwood City, CA, USA). Chromatographic separation was achieved on a Thermo BetaBasic C8 (50 mm × 2.1 mm, 3 μm, 150 Å) HPLC column. The sample was eluted in gradient elution mode using solvent A (0.1% formic acid in water) and solvent B (0.1% formic acid in acetonitrile, Sigma). The initial condition was 20% B for 1 min, followed by a linear gradient to 90% B by 4 min; from 5 to 6 min, 90% B was retained, and from 6 to 6.5 min, it was reverted back to the initial condition with 20% eluent B and retained from 6.5 to 9.0 min. Flowrate was set to 0.4 mL/min. The column temperature was 40 °C and the injection volume was 5μL. Nitrogen was used as the nebulizer gas (GS1), heater gas (GS2), and curtain gas with the optimum values set at 30, 30, and 35 (arbitrary units), respectively. Data were acquired in positive electrospray mode in the mass range of *m*/*z* 300 to 2500, with 1 s accumulation time. The source temperature was 350 °C, and the spray voltage was set to 5500 V. Declustering potential value was set to 80 V. PeakView Software TMV.2.2 (version2.2, Sciex, Redwood City, CA, USA) was used for deconvoluting the raw electrospray data to obtain the neutral molecular masses.

### 3.5. Thrombin Inhibitory Assay

Spectrophotometric enzyme tests were performed in transparent microtiter plates in a final volume of 200 µL. The reaction rates in the absence and in the presence of the inhibitor were measured. A total of 50 µL HEPES buffer (10 mM HEPES buffer (HEPES, Sigma); 150 mM NaCl, adjusted with 0,1 M NaOH to pH 7.5), 50 µL solution (2% DMSO in water) of different inhibitors at 400 µM (in case of measurement without inhibitor water), and 50 µL of thrombin solution (human thrombin, Sigma-Aldrich, 2 NIH (National Institute of Health Unit) E/mL) was pipetted into the microtiter plate. The plate was incubated for 30 min at 25 °C, and subsequently, 50 µL chromogenic substrate (S-2238 (H-DPhe-Pip-Arg-pNA 2HCl, Chromogenix), 160 µM) was added. The final concentration of the inhibitors was 100 µM, DMSO was 0.5%, thrombin was 0.5 NIH E/mL, and substrate was 40 µM. The microtiter plate was put into the spectrophotometer (Biotek H4), and the increase in absorbance at 405 nm at 25 °C was measured every 10 s. Change of absorbance form the initial, linear part of the curve was used to determine residual activity; measurements were carried out in triplicate in two independent experiments.

### 3.6. Quantum Chemical Calculations

The reactivity of compounds was also investigated by quantum chemical calculations. The energy barrier of the reaction between various non-methylated and methylated heterocycles and methyl thiolate was investigated. When a transitions state was not identified with methyl-thiolate, then cysteamine was used instead. Cysteamine in zwitterionic form was shown to be an appropriate reactant to model the reaction when thiolate attack and protonation both took place [28]. All calculations were performed with the Gaussian 09 program [25] using density functional theory (DFT) with the M062X [22] functional and 6-31+G** basis set with an IEFPCM implicit water model [24]. The def2SVP basis set [23] was used for molecules containing an iodine atom. The barriers were estimated as the difference of Gibbs free energies of activation between the transitions state and the separated reactants. All the geometries and transition states were optimized, and frequency calculations were made to assure that the structures were in a local minimum or in a saddle point, respectively.

## 4. Conclusions

We assembled the second generation of a heterocyclic covalent fragment library consisting of the *N*-quaternized analogues of small heterocyclic electrophiles. The methylated fragments were screened against MurA (UDP-N-acetylglucosamine 1-carboxyvinyltransferase, EC:2.5.1.7), resulting in several hits, including low-micromolar fragments, and the methylated heterocycles were found to consistently bind with larger affinities (5–100-fold) compared to their non-methylated analogues. The reactivity of the hits from the new derivatives was investigated against GSH in an NMR-based surrogate assay. Quantum chemical evaluation of the reaction barriers in model reactions provided an explanation of the enhanced reactivity. Covalent labelling of MurA was proven by LC–MS. The electrophilic nature of the hits identified from the library classified them as being PAINS active [29]. Therefore, GSH reactivity should be critically considered during the further optimization of these compounds. This strategy is in line with the observation that covalent inhibitors are typically drug-like compounds with crucial non-covalent interactions that reduce or even eliminate PAINS predicted promiscuity [30]. 

## Figures and Tables

**Table 1:** Comparison of the most active methylated heterocycles with their first-generation matched pairs.

Compound	MurA IC_50_ (µM) Non-Methylated [11]	MurA IC_50_ (µM) Methylated
**C3/C3+**	70	4.5
**C6/C6+**	408	64.7
**D3/D3+**	309	2.99
**F3/F3+**	3.8	4.8
**H3/H3+**	170	10.2
**Q2/Q2+**	77	2.9
fosfomycin 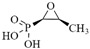	0.25 ± 0.01	-

**Table 2:** Gibbs free energies of activation for the reaction of non-methylated and methylated heterocycles with methyl-thiolate or cysteamine ^a^.

Compound	ΔG^#^ (kJ mol^−1^) *
**A1+**	41.0 (116.7)
**B1+**	71.9 (135.7)
**C1+**	56.2 (117.6)
**D1+**	5.2 (85.0)
**E1+**	12.2 (68.9)
**F1+**	21.6 (11.6)
**G1+**	0.3 (94.1)
**A2+**	23.9
**A3+**	0.0 ^b^
**A4+**	48.8 ^a^
**A5+**	32.9
**A6+**	51.9
**F3+**	0.0 ^b^
**F4+**	61.1 ^a^

^a^ Reaction modelled with zwitterionic cysteamine. ^b^ No barrier. * value for non-methylated pair in parentheses.

## Data Availability

Data is contained within the article and supplementary material.

## Supplementary Materials

The following supporting information can be downloaded at: https://www.mdpi.com/article/10.3390/ph15121484/s1, Figure S1: Deconvoluted mass spectra of the MurA labelled with fragment C3+; Table S1: Results of the biochemical assay against thrombin and the single-point GSH-reactivity assay. Compounds labelled by italic showed parallel reaction with the assay buffer; Table S2: Energy values for the computed structures.
