# Supplementary material for: Next-Generation Heterocyclic Electrophiles as Small-Molecule Covalent MurA Inhibitors
Source: Pharmaceuticals (Basel). 2022 Nov 29;15(12):1484. doi: 10.3390/ph15121484 (PMC9781958; doi:10.3390/ph15121484)
Supplement: Supplementary file 1 [file pharmaceuticals-15-01484-s001.zip › pharmaceuticals-2038715-supplementary.pdf]

Next-generation heterocyclic electrophiles as small molecule covalent MurA inhibitors

Péter Ábrányi-Balogh,[1] Aaron Keeley,[1] György G. Ferenczy,[1] László Petri,[1] Tímea Imre,[2] Katarina Grabrijan,[3] Martina Hrast,[3] Damijan Knez,[3] Janez Ilas,[3] Stanislav Gobec[3] and György Miklós Keserű[1,*]

[1] *Medicinal Chemistry Research Group, Research Centre for Natural Sciences, Magyar tudósok krt 2, H-1117 Budapest, Hungary*

[2] *MS Metabolomics Research Group, Research Centre for Natural Sciences, Magyar tudósok krt 2, H-1117 Budapest, Hungary*

[3] *Faculty of Pharmacy, University of Ljubljana, Askerceva 7, SI-1000, Ljubljana, Slovenia*

Table of contents

SUPPLEMENTARY TABLE S1. RESULTS OF THE BIOCHEMICAL ASSAY AGAINST THROMBIN AND THE SINGLE-POINT GSH-REACTIVITY ASSAY. COMPOUNDS LABELLED BY *ITALIC* SHOWED PARALLEL REACTION WITH THE ASSAY BUFFER. 2

FIGURE S1. DECONVOLUTED MASS SPECTRA OF THE MURA$_{EC}$ LABELLED WITH FRAGMENT C3+. 2

ALKYLATION OF HETEROCYCLIC ELECTROPHILES. PROCEDURE A 2

ALKYLATION OF HETEROCYCLIC ELECTROPHILES. PROCEDURE B 7

ENERGY VALUES FOR THE COMPUTED STRUCTURES 10

Supplementary Table S1. Results of the biochemical assay against thrombin and the single-point GSH-reactivity assay. Compounds labelled by *italic* showed parallel reaction with the assay buffer.

Compound	Residual activity (RA%) @ 1 mM thrombin after 30 min preincubation	Single point GSH reactivity (% conversion in 15 min, 16 equiv. GSH)
A1+	100%	100%
A3+	100%	100%
A5+	100%	100%
A6+	81.1%	88%
C3+	21.7%	100%
C6+	89.3%	68%
D3+	68.8%	*100%*
D6+	8.4%	*100%*
F3+	59.1%	*0%*
F4+	81.2%	N.A.
G3+	63.4%	100%
H3+	N.A.	100%
N3+	N.A.	*100%*
P3+	N.A.	*0%*
Q2+	80.5%	*100%*

Compounds labelled by *italic* showed parallel reaction with the assay buffer. N.A. stands for "Not available".

Figure S1. Deconvoluted mass spectra of the $MurA_{EC}$ labelled with fragment C3+.

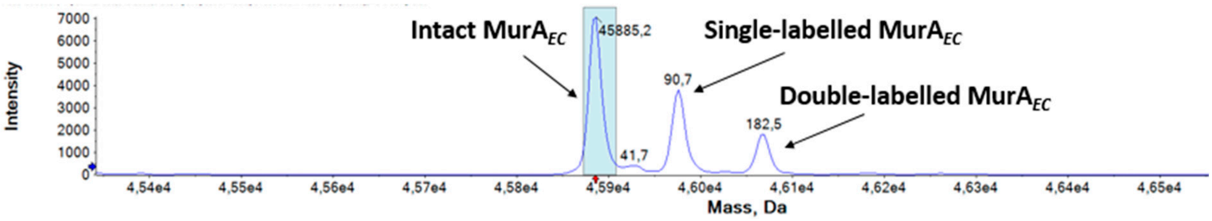

Alkylation of heterocyclic electrophiles. Procedure A

To a solution of appropriate heterocycle (1.0 equiv.) in acetonitrile under argon, was added CH_3I (2.0 equiv.). For the di-substitution of imidazoles and pyrazoles (4.0 equiv.) was added. The mixture was heated at 90 °C for 24 h, and then cooled to room temperature. After passing through a celite pad, washed with additional MeCN, a solvent was removed under reduced pressure. The crude product was purified by recrystallization in CH_3CN/EtOAc if needed.

General Procedure **A** afforded

A1+: 2-chloro-1-methylpyridin-1-ium iodide (0.74 mmol, 190 mg, 65%) as a white solid. ^{1}H NMR (300 MHz, DMSO-d_6) δ 9.25 (d, *J* = 5.7 Hz, 1H), 8.67 (d, *J* = 7.8 Hz, 1H), 8.15 (t, *J* = 7.3 Hz, 1H), 8.07 (t, *J* = 6.3 Hz, 1H), 4.41 (s, 3H) ppm. LC-MS: t_R = 0.7 min, 100% by UV, [M+H$^+$] found: 128.0.

A2+: 2-bromo-1-methylpyridin-1-ium iodide (0.48 mmol, 145 mg, 48%) as a pale yellow solid. ^{1}H NMR (300 MHz, D_2O) δ 8.92 (d, *J* = 5.5 Hz, 1H), 8.50 (d, *J* = 9.1 Hz, 1H), 7.97 (t, *J* = 8.5 Hz, 1H), 7.89 (t, *J* = 6.3 Hz, 1H), 4.38 (s, 3H) ppm. LC-MS: t_R = 0.74 min, 100% by UV, [M+H$^+$] found: 173.0.

A3+: 2-iodo-1-methylpyridin-1-ium iodide (0.68 mmol, 235 mg, 68%) as a pale yellow solid. ^{1}H NMR (300 MHz, d_2o) δ 8.91 (d, *J* = 7.9 Hz, 1H), 8.50 (d, *J* = 10.7 Hz, 1H), 7.97 (t, *J* = 8.4 Hz, 1H), 7.87 (t, *J* = 10.4 Hz, 1H), 4.38 (s, 3H) ppm. LC-MS : t_R = solvent front, 100% by NMR, [M+H$^+$] found: 220.0

A4+: 2-cyano-1-methylpyridin-1-ium iodide (0.28 mmol, 69 mg, 28%) as a yellow solid. ^{1}H NMR (300 MHz, DMSO-d_6) δ 9.37 (d, *J* = 5.9 Hz, 1H), 8.83 (q, *J* = 7.5 Hz, 2H), 8.47 (dd, *J* = 8.9, 4.1 Hz, 1H), 4.52 (s, 3H) ppm. LC-MS: t_R = 0.7 min, 100% by UV, [M+H$^+$] found: 119.0.

A5+: 1-methyl-2-vinylpyridin-1-ium iodide (0.34 mmol, 84 mg, 34%) as a yellow solid. ^{1}H NMR (500 MHz, DMSO-d_6) δ 8.93 (d, *J* = 6.0 Hz, 1H), 8.53 (t, *J* = 7.6 Hz, 1H), 8.34 (d, *J* = 7.0 Hz, 1H), 7.98 (t, *J* = 6.9 Hz, 1H), 7.22 (dd, *J* = 17.1, 11.4 Hz, 1H), 6.47 (d, *J* = 17.1 Hz, 1H), 6.13 (d, *J* = 11.3 Hz, 1H), 4.27 (s, 3H) ppm. LC-MS: t_R = 0.7 min, 100% by UV, [M+H$^+$] found: 120.0.

A6+: 2-ethynyl-1-methylpyridin-1-ium iodide (0.22 mmol, 53 mg, 22%) as a pink solid. ^{1}H NMR (300 MHz, DMSO-d_6) δ 9.12 (d, *J* = 6.0 Hz, 1H), 8.59 (t, *J* = 7.9 Hz, 1H), 8.35 (d, *J* = 7.9 Hz, 1H), 8.14 (t, *J* = 6.8 Hz, 1H), 5.88 (s, 1H), 4.36 (s, 3H) ppm. LC-MS: t_R = solvent front, 100% by NMR, [M+H$^+$] found: 118.0.

B1+: 3-chloro-1-methylpyridin-1-ium iodide (0.19 mmol, 49 mg, 19%) as a yellow solid. ^{1}H NMR (300 MHz, DMSO-d_6) δ 9.40 (s, 1H), 9.01 (d, *J* = 5.9 Hz, 1H), 8.77 (d, *J* = 8.4 Hz, 1H), 8.22 – 8.15 (m, 1H), 4.35 (s, 3H) ppm. LC-MS: t_R = 0.6 min, 100% by UV, [M+H$^+$] found: 128.0.

B2+: 3-bromo-1-methylpyridin-1-ium iodide (0.55 mmol, 166 mg, 55%) as a yellow solid. ^{1}H NMR (300 MHz, DMSO-d_6) δ 9.43 (s, 1H), 9.02 (d, *J* = 5.9 Hz, 1H), 8.86 (d, *J* = 8.4 Hz, 1H), 8.14 – 8.05 (m, 1H), 4.33 (s, 3H) ppm. LC-MS: t_R = 0.76 min, 100% by UV, [M+H$^+$] found: 173.0.

B3+: 3-iodo-1-methylpyridin-1-ium iodide (0.47 mmol, 163 mg, 72%) as a yellow solid. ^{1}H NMR (300 MHz, D_2O) δ 9.08 (s, 1H), 8.77 (d, *J* = 8.3 Hz, 1H), 8.71 (d, *J* = 6.0 Hz, 1H), 7.69 (t, 1H), 4.25 (s, 3H) ppm. LC-MS: t_R = 0.7 min, 100% by UV, [M+H$^+$] found: 220.0.

B4+: 3-cyano-1-methylpyridin-1-ium iodide (0.85 mmol, 210 mg, 87%) as a yellow solid. ^{1}H NMR (300 MHz, DMSO-d_6) δ 9.74 (s, 1H), 9.25 (d, *J* = 6.1 Hz, 1H), 9.08 (d, *J* = 8.1 Hz, 1H), 8.39 – 8.29 (m, 1H), 4.37 (s, 3H) ppm. LC-MS: t_R = solvent front, 100% by NMR, [M+H$^+$] found: 119.0.

B5+: 1-methyl-3-vinylpyridin-1-ium iodide (0.68 mmol, 167 mg, 84%) as a yellow solid. m.p. 78 °C, ^{1}H NMR (300 MHz, D_2O) δ 8.74 (s, 1H), 8.53 (d, *J* = 5.8 Hz, 1H), 8.46 (d, *J* = 8.2 Hz, 1H), 7.88 (t, *J* = 6.8 Hz, 1H), 6.75 (dt, *J* = 19.1, 9.6 Hz, 1H), 6.06 (d, *J* = 17.6 Hz, 1H), 5.64 (d, *J* = 11.1 Hz, 1H), 4.28 (s, 3H) ppm. LC-MS: t_R = 0.7 min, 100% by UV, [M+H$^+$] found: 120.0

B6+: 3-ethynyl-1-methylpyridin-1-ium iodide (0.35 mmol, 86 mg, 35%) as a brown solid. ^{1}H NMR (300 MHz, DMSO-d_6) δ 9.29 (s, 1H), 9.00 (d, *J* = 6.0 Hz, 1H), 8.68 (d, *J* = 8.1 Hz, 1H), 8.19 – 8.09 (m, 1H), 4.97 (s, 1H), 4.33 (s, 3H) ppm. LC-MS: t_R = solvent front, 100% by NMR, [M+H$^+$] found: 118.0.

C1+: 4-chloro-1-methylpyridin-1-ium iodide (0.74 mmol, 190 mg, 65%) as a brown solid. ^{1}H-NMR (300 MHz, D_2O) δ 8.73 (d, *J* = 6.8 Hz, 2H), 8.10 (d, *J* = 9.0 Hz, 2H), 4.34 (s, 3H) ppm. LC-MS: t_R = 0.75 min, 100% by UV, [M+H$^+$] found: 128.0.

C2+: 4-bromo-1-methylpyridin-1-ium iodide (0.97 mmol, 290 mg, 97%) as a black solid. ^{1}H NMR (300 MHz, DMSO-d_6) δ 8.67 (d, *J* = 6.2 Hz, 2H), 8.59 (d, *J* = 6.2 Hz, 2H), 4.23 (s, 3H) ppm. LC-MS: t_R = 0.8 min, 100% by UV, [M+H$^+$] found: 173.0.

C3+: 4-iodo-1-methylpyridin-1-ium iodide (0.50 mmol, 175 mg, 50%) as a black solid. ^{1}H NMR (300 MHz, DMSO-d_6) δ 8.64 (d, *J* = 6.6 Hz, 1H), 8.60 (d, *J* = 6.6 Hz, 1H), 4.22 (s, 1H) ppm. LC-MS: t_R = 0.6 min, 100% by UV, [M+H$^+$] found: 220.0.

C4+: 4-cyano-1-methylpyridin-1-ium iodide (0.66 mmol, 163 mg, 84%) as a yellow solid. ^{1}H NMR (300 MHz, DMSO-d_6) δ 9.28 (d, *J* = 6.4 Hz, 2H), 8.69 (d, *J* = 5.6 Hz, 2H), 4.42 (s, 3H) ppm. LC-MS: t_R = solvent front, 100% by NMR, [M+H$^+$] found: 119.0.

D3+: 2-iodo-1-methylpyrimidin-1-ium iodide (0.06 mmol, 22 mg, 20%) as a white solid. m.p. 125 °C, ^{1}H NMR (300 MHz, D_2O) δ 9.07 (d, *J* = 8.7 Hz, 1H), 7.88 (d, *J* = 13.6 Hz, 1H), 5.73 – 5.60 (m, 1H), 2.70 (s, 3H) ppm. ^{13}C NMR (75 MHz, DMSO-d_6) δ 162.2, 162.0, 158.1, 150.6, 48.4 ppm. HRMS (ESI): (M+H)$^+$ calcd. for $C_5H_6IN_2^+$, 220.9570; found, 220.9581. LC-MS: t_R = solvent front, 95% by NMR, [M+H$^+$] found: 221.0.

F1+: 5-chloro-1-methylpyrimidin-1-ium iodide (0.12 mmol, 32 mg, 23%) as a yellow solid. m.p. 133 °C, ^{1}H NMR (300 MHz, DMSO-d_6) δ 9.80 (s, *J* = 3.0 Hz, 1H), 9.64 (s, *J* = 2.0 Hz, 1H), 4.28 (s, 3H) ppm. ^{13}C NMR (126 MHz, DMSO-d_6) δ 154.9, 151.5, 150.1, 118.0, 44.5 ppm. HRMS (ESI): (M+H)$^+$ calcd. for $C_5H_6ClN_2^+$, 129.0214; found, 129.0228. LC-MS: t_R = solvent front, 90% by NMR, [M+H$^+$] found: 130.0.

F2+: 5-bromo-1-methylpyrimidin-1-ium iodide (0.56 mmol, 168 mg, 68%) as a yellow solid. m.p. 196 °C, ^{1}H NMR (500 MHz, DMSO-d_6) δ 9.85 (s, 1H), 9.82 (s, 1H), 9.66 (s, 1H), 4.27 (s, 3H) ppm. ^{13}C NMR (126 MHz, DMSO-d_6) δ 164.9, 154.5, 153.0, 120.0, 45.6 ppm. HRMS (ESI): (M+H)$^+$ calcd. for $C_5H_6BrN_2^+$, 172.9709; found, 172.9791. LC-MS: t_R = solvent front, 90% by NMR, [M+H$^+$] found: 174.0.

F3+: 5-iodo-1-methylpyrimidin-1-ium iodide (0.86 mmol, 298 mg, 86%) as a yellow solid. m.p. 182 °C, ^{1}H NMR (300 MHz, DMSO-d_6) δ 9.77 (s, 2H), 9.70 (s, 1H), 4.21 (s, 3H) ppm. ^{13}C NMR (75 MHz, DMSO-d_6) δ 168.9, 158.0, 152.9, 96.6, 45.4 ppm. HRMS (ESI): (M+H)$^+$ calcd. for $C_5H_6IN_2^+$, 220.9570; found, 220.9564. LC-MS: t_R = solvent front, 100% by NMR, [M+H$^+$] found: 221.0.

F5+: 1-methyl-5-vinylpyrimidin-1-ium iodide (0.13 mmol, 31 mg, 76%) as a brown solid. m.p. 125 °C, ^{1}H NMR (300 MHz, DMSO-d_6) δ 9.66 (s, 1H), 9.57 (s, 2H), 6.90 (dd, *J* = 17.8, 11.2 Hz, 1H), 6.40 (d, *J* = 17.8 Hz, 1H), 5.87 (d, *J* = 11.2 Hz, 1H), 4.27 (s, 3H) ppm. ^{13}C NMR (75 MHz, DMSO-d_6) δ 165.9, 164.4, 163.8, 156.0, 152.9, 96.6, 45.4 ppm. HRMS (ESI): (M+H)$^+$ calcd. for $C_7H_9N_2^+$, 121.0760; found, 121.0758. LC-MS: t_R = solvent front, 100% by NMR, [M+H$^+$] found: 121.0.

G1+: 3-chloro-1-methylpyrazin-1-ium iodide (0.15 mmol, 39 mg, 25%) as a brown solid. m.p. 168 °C, ^{1}H NMR (300 MHz, DMSO-d_6) δ 9.62 (s, 1H), 9.35 (d, *J* = 3.2 Hz, 1H), 9.19 (d, *J* = 2.7 Hz, 1H), 4.38 (s, 3H) ppm. LC-MS: t_R = solvent front, 90% by NMR, [M+H$^+$] found: 130.0.

G2+: 3-bromo-1-methylpyrazin-1-ium iodide (0.22 mmol, 67 mg, 27%) as a yellow solid. m.p. 192 °C, ^{1}H NMR (300 MHz, DMSO-d_6) δ 9.63 (s, 1H), 9.28 (br s, 1H), 9.15 (br s, 1H), 4.28 (s, 3H) ppm. ^{13}C NMR (126 MHz, DMSO-d_6) δ 148.5, 143.9, 136.6, 121.8, 48.2 ppm. HRMS (ESI): (M+H)$^+$ calcd. for $C_5H_6BrN_2^+$, 172.9709; found, 172.9712. LC-MS: t_R = 0.7 min, 100% by UV, [M+H$^+$] found: 174.0.

G3+: 3-iodo-1-methylpyrazin-1-ium iodide (0.14 mmol, 50 mg, 24%) as a yellow solid. m.p. 210 °C, ^{1}H NMR (500 MHz, DMSO-d_6) δ 9.61 (s, 1H), 9.26 (br s, 1H), 9.14 (d, *J* = 2.7 Hz, 1H), 4.28 (s, 3H) ppm. ^{13}C NMR (126 MHz, DMSO-d_6) δ 151.2, 145.9, 137.0, 122.0, 48.6 ppm. HRMS (ESI): (M+H)$^+$ calcd. for $C_5H_6IN_2^+$, 220.9570; found, 220.9582. LC-MS: t_R = 0.75 min, 100% by UV, [M+H$^+$] found: 221.0.

H1+: 2-chloro-1,3-dimethyl-1H-imidazol-3-ium iodide (0.36 mmol, 92 mg, 89%) as a pale yellow solid. ^{1}H NMR (500 MHz, DMSO-d_6) δ 7.81 (s, 2H), 3.79 (s, 6H) ppm. LC-MS: t_R = solvent front, 100% by NMR, [M+H$^+$] found: 132.0.

H2+: 2-bromo-1,3-dimethyl-1H-imidazol-3-ium iodide (0.32 mmol, 98 mg, 98%) as a yellow solid. ^{1}H NMR (500 MHz, DMSO-d_6) δ 7.87 (s, 2H), 3.80 (s, 6H) ppm. LC-MS: t_R = solvent front, 100% by NMR, [M+H$^+$] found: 176.0.

H3+: 2-iodo-1,3-dimethyl-1H-imidazol-3-ium iodide (0.25 mmol, 89 mg, 89%) as a brown solid. ^{1}H NMR (400 MHz, CD_3CN) δ 7.60 (s, 2H), 3.87 –3.72 (m, 6H) ppm. LC-MS: t_R = solvent front, 100% by NMR, [M+H$^+$] found: 223.0.

H4+: 2-cyano-1,3-dimethyl-1H-imidazol-3-ium iodide (0.23 mmol, 57 mg, 57%) as a white solid. ^{1}H NMR (300 MHz, DMSO-d_6) δ 7.84 (s, 2H), 3.85 (s, 3H), 3.82 (s, 3H) ppm. LC-MS: t_R = solvent front, 100% by NMR, [M+H$^+$] found: 122.0.

I1+: 4-chloro-1,3-dimethyl-1H-imidazol-3-ium iodide (0.80 mmol, 205 mg, 80%) as a pale yellow solid. ^{1}H NMR (300 MHz, DMSO-d_6) δ 9.19 (s, 1H), 8.01 (s, 1H), 3.85 (s, 3H), 3.32 (s, 3H) ppm. LC-MS: t_R = solvent front, 100% by NMR, [M+H$^+$] found: 132.0.

I2+: 4-bromo-1,3-dimethyl-1H-imidazol-3-ium iodide (0.96 mmol, 290 mg, 95%) as a yellow solid. ^{1}H NMR (300 MHz, DMSO-d_6) δ 9.22 (s, 1H), 8.00 (s, 1H), 3.85 (s, 3H), 3.78 (s, 3H) ppm. LC-MS: t_R = solvent front, 100% by NMR, [M+H$^+$] found: 176.0.

I3+: 4-iodo-1,3-dimethyl-1H-imidazol-3-ium iodide (0.05 mmol, 17 mg, 17%) as a white solid. m.p. 210 ^{o}C, ^{1}H NMR (300 MHz, DMSO-d_6) δ 9.20 (s, 1H), 7.91 (br s, 1H), 3.83 (s, 3H), 3.75 (s, 3H) ppm. LC-MS: t_R = solvent front, 100% by NMR, [M+H$^+$] found: 223.0.

I4+: 4-cyano-1,3-dimethyl-1H-imidazol-3-ium iodide (0.27 mmol, 66 mg, 66%) as a brown solid. ^{1}H NMR (300 MHz, D_2O) δ 9.44 (s, 1H), 8.14 (s, 1H), 3.83 (s, 3H), 3.78 (s, 3H) ppm. LC-MS: t_R = solvent front, 100% by NMR, [M+H$^+$] found: 122.0.

K2+: 5-bromo-1,2-dimethyl-1H-pyrazol-2-ium iodide (0.60 mmol, 180 mg, 59%) as a white solid. m.p. 115 ^{o}C, ^{1}H NMR (300 MHz, DMSO-d_6) δ 8.56 (d, *J* = 2.6 Hz, 1H), 7.19 (d, *J* = 2.8 Hz, 1H), 4.15 (s, 3H), 4.04 (s, 3H) ppm. ^{13}C NMR (126 MHz, DMSO-d_6) δ 156.3, 140.3, 128.7, 42.7, 36.2 ppm. HRMS (ESI): (M+H)$^+$ calcd. for $C_5H_8BrN_2^+$, 174.9865; found, 174.9868. LC-MS : t_R = solvent front, 85% by NMR, [M+H$^+$] found: 176.0.

K3+: 5-iodo-1,2-dimethyl-1H-pyrazol-2-ium iodide (0.10 mmol, 34 mg, 34%) as a pale yellow solid. ^{1}H NMR (300 MHz, DMSO-d_6) δ 8.46 (d, *J* = 2.6 Hz, 1H), 7.15 (d, *J* = 2.9 Hz, 1H), 4.18 (s, 3H), 4.08 (s, 3H) ppm. LC-MS: t_R = solvent front, 100% by NMR, [M+H$^+$] found: 223.0.

K5+: 1,2-dimethyl-5-vinyl-1H-pyrazol-2-ium iodide (0.5 mmol, 111mg, 44%) as a yellow solid. m.p. 160 ^{o}C, ^{1}H NMR (300 MHz, DMSO-d_6) δ 8.43 (d, *J* = 2.7 Hz, 1H), 7.18 (d, *J* = 2.9 Hz, 1H), 6.98 (dd, *J* = 17.3, 11.2 Hz, 1H), 6.33 (d, *J* = 17.3 Hz, 1H), 5.91 (d, *J* = 11.2 Hz, 1H), 4.09 (s, 3H), 4.05 (s, 3H) ppm. ^{13}C NMR (126 MHz, DMSO-d_6) δ 146.3, 137.3, 126.7, 122.1, 104.2, 37.5, 34.3 ppm. HRMS (ESI): (M+H)$^+$ calcd. for $C_7H_{11}N_2^+$, 123.0917; found, 123.0921. LC-MS: t_R = solvent front, 100% by NMR, [M+H$^+$] found: 123.0.

K6+: 5-ethynyl-1,2-dimethyl-1H-pyrazol-2-ium iodide (0.24 mmol, 60mg, 30%) as a black solid. ^{1}H NMR (300 MHz, DMSO-d_6) δ 8.58 (d, *J* = 2.7 Hz, 1H), 7.20 (d, *J* = 2.8 Hz, 1H), 5.48 (s, 1H), 4.11 (s, 3H), 4.06 (s, 3H) ppm. LC-MS: t_R = solvent front, 85% by NMR, [M+H$^+$] found: 121.0.

L1+: 4-chloro-1,2-dimethyl-1H-pyrazol-2-ium iodide (0.21 mmol, 53 mg, 54%) as a white solid. m.p. 285 °C, ^{1}H NMR (300 MHz, DMSO-d_6) δ 8.78 (s, 2H), 4.09 (s, 6H) ppm. ^{13}C NMR (75 MHz, DMSO-d_6) δ 135.9, 110.1, 37.9 ppm. HRMS (ESI): (M+H)$^+$ calcd. for $C_5H_8ClN_2^+$, 131.0371; found, 131.0382. LC-MS: t_R = solvent front, 100% by NMR, [M+H$^+$] found: 132.0.

L2+: 4-bromo-1,2-dimethyl-1H-pyrazol-2-ium iodide (0.17 mmol, 51 mg, 51%) as a white solid. m.p. 216 °C, ^{1}H NMR (300 MHz, DMSO-d_6) δ 8.76 (s, 2H), 4.09 (s, 6H) ppm. ^{13}C NMR (75 MHz, DMSO-d_6) δ 137.9, 93.8, 37.9 ppm. HRMS (ESI): (M+H)$^+$ calcd. for $C_5H_8BrN_2^+$, 174.9865; found, 174.9882. LC-MS: t_R = solvent front, 100% by NMR, [M+H$^+$] found: 176.0.

L3+: 4-iodo-1,2-dimethyl-1H-pyrazol-2-ium iodide (0.14 mmol, 51 mg, 51%) as a white solid. m.p. 155 °C, ^{1}H NMR (300 MHz, DMSO-d_6) δ 8.67 (s, 2H), 4.09 (s, 6H) ppm. ^{13}C NMR (75 MHz, DMSO-d_6) δ 146.9, 93.8, 37.9 ppm. HRMS (ESI): (M+H)$^+$ calcd. for $C_5H_8IN_2^+$, 222.9727; found, 222.9732. LC-MS: t_R = solvent front, 100% by NMR, [M+H$^+$] found: 223.0.

L6+: 4-ethynyl-1,2-dimethyl-1H-pyrazol-2-ium iodide (1 mmol, 250mg, 99%) as a brown solid. ^{1}H NMR (300 MHz, DMSO-d_6) δ 8.79 (s, 2H), 4.46 (s, 1H), 4.09 (s, 6H) ppm. LC-MS: t_R = solvent front, 100% by NMR, [M+H$^+$] found: 121.0.

P3+: 4-iodo-2,3,5-trimethylisoxazol-2-ium iodide (0.24 mmol, 89mg, 24%) as a brown solid. ^{1}H NMR (300 MHz, D_2O) δ 4.20 (s, 3H), 2.56 (s, 3H), 2.54 (s, 3H) ppm. LC-MS: t_R = solvent front, 100% by NMR, [M+H$^+$] found: 238.0.

Q2+: 5-bromo-3-methylthiazol-3-ium iodide (0.41 mmol, 124 mg, 41%) as a brown solid. ^{1}H NMR (300 MHz, DMSO-d_6) δ 10.10 (s, 1H), 8.73 (br s, 1H), 4.16 (s, 3H) ppm. LC-MS: t_R = solvent front, 100% by NMR, [M+H$^+$] found: 179.0.

R2+: 2-bromo-3-methylthiazol-3-ium iodide (0.20 mmol, 59 mg, 20%) as a brown solid. ^{1}H NMR (300 MHz, DMSO-d_6) δ 8.88 (d, J = 3.6 Hz, 1H), 8.85 (d, J = 3.6 Hz, 1H), 4.12 (s, 3H) ppm. LC-MS: t_R = solvent front, 100% by NMR, [M+H$^+$] found: 179.0.

Alkylation of heterocyclic electrophiles. Procedure B

A solution of heterocycle (1.0 equiv.) in dry dichloromethane or chloroform was cooled to 0 °C under argon atmosphere. Methyl trifluoromethanesulfonate (1.2 equiv.) was added dropwise. For the di-substitution of imidazoles and pyrazoles (2.4 equiv.) was added. The reaction mixture was allowed to stir until all the starting materials disappeared followed by TLC and HPLC-MS. Precipitated solid was collected by filtration or when the product was a liquid, the reaction mixture was evaporated. The crude product was purified by dissolving in small amount of dry acetonitrile and precipitation by the

addition of dry diethyl ether (2 times). The final product was washed with small amount of dry diethyl ether and dried *in vacuo*.

General Procedure **B** afforded

C5+: 1-methyl-4-vinylpyridin-1-ium trifluoromethanesulfonate (0.44 mmol, 119 mg, 48%) as a yellow solid. ^{1}H NMR (300 MHz, DMSO-d$_6$) δ 9.64 (d, *J* = 10.2 Hz, 2H), 9.30 (d, *J* = 15.6 Hz, 2H), 6.89 (dd, *J* = 17.7, 11.1 Hz, 1H), 6.30 (d, *J* = 17.5 Hz, 1H), 5.75 (d, *J* = 11.2 Hz, 1H), 4.34 (s, 3H) ppm. LC-MS: t_R = 0.74 min, 100% by UV, [M+H$^+$] found: 120.0.

C6+: 4-ethynyl-1-methylpyridin-1-ium trifluoromethanesulfonate (0.09 mmol, 24 mg, 46%) as a black solid. ^{1}H NMR (300 MHz, DMSO-d$_6$) δ 9.00 (d, *J* = 6.6 Hz, 2H), 8.22 (d, *J* = 6.5 Hz, 2H), 5.41 (s, 1H), 4.33 (s, 3H) ppm. LC-MS: t_R = solvent front, 100% by NMR, [M+H$^+$] found: 118.0.

D1+: 2-chloro-1-methylpyrimidin-1-ium trifluoromethanesulfonate (0.43 mmol, 120 mg, 57%) as a brown solid. ^{1}H NMR (500 MHz, DMSO-d$_6$) δ 9.04 (dd, *J* = 6.2, 2.5 Hz, 1H), 8.81 (dd, *J* = 5.9, 2.6 Hz, 1H), 6.95 (t, *J* = 6.0 Hz, 1H), 3.64 (s, 3H) ppm. LC-MS: t_R = solvent front, 100% by NMR, [M+H$^+$] found: 130.0.

D2+: 2-bromo-1-methylpyrimidin-1-ium trifluoromethanesulfonate (0.64 mmol, 206 mg, 69%) as a brown solid. ^{1}H NMR (500 MHz, DMSO-d$_6$) δ 8.62 (br s, 1H), 8.46 (br s, 1H), 6.58 (t, *J* = 5.4 Hz, 1H), 3.49 (s, 3H) ppm. LC-MS: t_R = solvent front, 85% by NMR, [M+H$^+$] found: 174.0.

D6+: 2-ethynyl-1-methylpyrimidin-1-ium trifluoromethanesulfonate (0.77 mmol, 208 mg, 70%) as a black solid. m.p. 77 ^{o}C, ^{1}H NMR (300 MHz, DMSO-d$_6$) δ 9.43 (dd, *J* = 8.5, 5.7 Hz, 2H), 8.27 (t, *J* = 5.6 Hz, 1H), 5.92 (s, 1H), 4.33 (s, 3H) ppm. ^{13}C NMR (75 MHz, DMSO-d$_6$) δ 164.2, 155.0, 123.2, 96.7, 75.2, 55.9, 46.9 ppm. HRMS (ESI): (M+H)$^+$ calcd. for $C_7H_7N_2^+$, 119.0604; found, 119.0610. LC-MS: t_R = solvent front, 100% by NMR, [M+H$^+$] found: 119.0.

E1+: 4-chloro-1-methylpyrimidin-1-ium trifluoromethanesulfonate (0.03 mmol, 8 mg, 23%) as a yellow solid. m.p. 54 ^{o}C, ^{1}H NMR (500 MHz, DMSO-d$_6$) δ 8.77 (s, 1H), 8.01 (d, *J* = 7.2 Hz, 1H), 6.52 (d, *J* = 7.2 Hz, 1H), 4.19 (s, 3H) ppm. ^{13}C NMR (126 MHz, DMSO-d$_6$) δ 152.3, 150.0, 148.6, 117.0. 43.0 ppm. HRMS (ESI): (M+H)$^+$ calcd. for $C_5H_6ClN_2^+$, 129.0214; found, 129.0220. LC-MS: t_R = solvent front, 85% by NMR, [M+H$^+$] found: 130.0.

E4+: 4-cyano-1-methylpyrimidin-1-ium trifluoromethanesulfonate (0.70 mmol, 188 mg, 76%) as a brown solid. ^{1}H NMR (300 MHz, DMSO-d$_6$) δ 7.8 (d, *J* = 7.6 Hz, 1H), 7.1 (s, 1H), 6.2 (d, *J*=7.6 Hz 1H), 3.7 (s, 3H) ppm. LC-MS: t_R = solvent front, 100% by NMR, [M+H$^+$] found: 120.0.

F6+: 5-ethynyl-1-methylpyrimidin-1-ium trifluoromethanesulfonate (0.38 mmol, 104 mg, 35%) as a brown solid. m.p. 190 ^{o}C, ^{1}H NMR (300 MHz, DMSO-d$_6$) δ 9.64 (s, 1H), 9.55 (br s, 2H), 5.40 (s, 1H), 4.30

(s, 3H) ppm. ^{13}C NMR (75 MHz, DMSO-d_6) δ 169.0, 158.0, 152.9, 96.6, 72.1, 54.9, 45.4 ppm. HRMS (ESI): $(M+H)^+$ calcd. for $C_7H_7N_2^+$, 119.0604; found, 119.0609. LC-MS: t_R = solvent front, 100% by NMR, [M+H$^+$] found: 119.0.

G4+: 3-cyano-1-methylpyrazin-1-ium trifluoromethanesulfonate (1.11 mmol, 300 mg, 56%) as a white solid. m.p. 110 °C, ^1H NMR (500 MHz, DMSO-d_6) δ 9.95 (s, 1H), 9.66 (br s, 1H), 9.46 (d, *J* = 3.2 Hz, 1H), 4.44 (s, 3H) ppm. ^{13}C NMR (126 MHz, DMSO-d_6) δ 151.5, 133.9, 122.2, 119.8, 114.2, 49.7 ppm. HRMS (ESI): $(M+H)^+$ calcd. for $C_6H_6N_3^+$, 120.0556; found, 120.0564. LC-MS: t_R = solvent front, 100% by NMR, [M+H$^+$] found: 120.0.

G5+: 1-methyl-3-vinylpyrazin-1-ium trifluoromethanesulfonate (0.54 mmol, 147 mg, 59%) as a brown solid. m.p. 179 °C, ^1H NMR (300 MHz, D_2O) δ 9.18 (s, 1H), 8.96 (br s, 1H), 8.67 (br s, 1H), 7.03 – 6.90 (m, 1H), 6.52 (d, *J* = 17.5 Hz, 1H), 5.94 (d, *J* = 10.9 Hz, 1H), 4.39 (s, 3H) ppm. ^{13}C NMR (126 MHz, DMSO-d_6) δ 150.8, 144.8, 144.2, 143.6, 134.1, 121.2. 50.4 ppm. HRMS (ESI): $(M+H)^+$ calcd. for $C_7H_9N_2^+$, 121.0760; found, 121.0792. LC-MS: t_R = solvent front, 90% by NMR, [M+H$^+$] found: 121.0.

G6+: 3-ethynyl-1-methylpyrazin-1-ium trifluoromethanesulfonate (0.12 mmol, 33 mg, 41%) as a black solid. m.p. 186 °C, ^1H NMR (300 MHz, d_2o) δ 9.28 (s, 1H), 9.14 (br s, 1H), 8.91 (br s, 1H), 4.42 (s, 3H) ppm. ^{13}C NMR (126 MHz, DMSO-d_6) δ 150.0, 143.4 139.2, 138.7, 86.8, 72.9, 48.6 ppm. HRMS (ESI): $(M+H)^+$ calcd. for $C_7H_7N_2^+$, 119.0604; found, 119.0719. LC-MS: t_R = solvent front, 85% by NMR, [M+H$^+$] found: 119.0.

L4+: 4-cyano-1,2-dimethyl-1H-pyrazol-2-ium trifluoromethanesulfonate (0.24 mmol, 65mg, 51%) as a brown solid. m.p. 155 °C, ^1H NMR (300 MHz, d_2o) δ 8.77 (s, 2H), 4.12 (s, 6H) ppm. ^{13}C NMR (75 MHz, DMSO-d_6) δ 145.9, 128.1, 105.3, 38.3 ppm. HRMS (ESI): $(M+H)^+$ calcd. for $C_6H_8N_3^+$, 122.0713; found, 122.0725. LC-MS: t_R = solvent front, 85% by NMR, [M+H$^+$] found: 122.0.

N3+: 2-iodo-3-methyloxazol-3-ium trifluoromethanesulfonate (0.25 mmol, 88mg, 82%) as a white solid. m.p. 148 °C, ^1H NMR (500 MHz, DMSO-d_6) δ 7.13 (d, *J* = 2.0 Hz, 1H), 7.02 (d, *J* = 2.0 Hz, 1H), 3.11 (s, 3H) ppm. ^{13}C NMR (126 MHz, DMSO-d_6) δ 156.6, 136.5, 130.7, 37.2 ppm. HRMS (ESI): $(M+H)^+$ calcd. for $C_4H_5INO^+$, 209.9410; found, 209.9421. LC-M : t_R = solvent front, 95% by NMR, [M+H$^+$] found: 210.0.

R4+: 2-cyano-3-methylthiazol-3-ium trifluoromethanesulfonate (0.80 mmol, 220 mg, 53%) as a white solid. m.p. 125 °C, ^1H NMR (300 MHz, DMSO-d_6) δ 8.78 (d, *J* = 3.5 Hz, 1H), 8.70 (d, *J* = 3.5 Hz, 1H), 4.31 (s, 3H) ppm. ^{13}C NMR (126 MHz, DMSO-d_6) δ 151.6, 141.5, 132.7, 106.4, 42.2 ppm. HRMS (ESI): $(M+H)^+$ calcd. for $C_5H_5N2S^+$, 125.0168; found, 125.0170. LC-MS: t_R = solvent front, 95% by NMR, [M+H$^+$] found: 125.0.

Table S2. Energy values for the computed structures

E stands for energy, ZPE for zero point energy, U for internal energy, H for enthalpy, G for Gibbs free energy, S for entropy.

File name	E	ZPE	U	H	G	S
Cl-_m062x631+dp_h2o_f.log	-460.3	-460.3	-460.3	-460.3	-460.4	36.6
MeS-_m062x631+dp_h2o.log	-438.2	-438.1	-438.1	-438.1	-438.2	55.9
cysteamine.log	-533.3	-533.2	-533.2	-533.2	-533.2	71.5
2bromopyridineMe+.log	-2859.1	-2859.0	-2859.0	-2859.0	-2859.0	85.2
2cyanopyridineMe+.log	-380.1	-380.0	-380.0	-380.0	-380.0	86.2
2vinylpyridineMe+.log	-365.3	-365.1	-365.1	-365.1	-365.2	87.1
2ethynylpyridineMe+.log	-364.0	-363.9	-363.9	-363.9	-363.9	86.5
5cyanopyrimidineMe+.log	-396.2	-396.0	-396.0	-396.0	-396.1	87.9
2bromopyridineMe+_Cl-_MeS-_m062x631+dp_h2o_QST3.log	-3757.645564	-3757.486385	-3757.473137	-3757.472193	-3757.529326	120.246
2cyanopyridineMe+_Cysteamine_m062x631+dp_h2o_QST2.log	-913.3910462	-913.16155	-913.148301	-913.147356	-913.202072	115.159
2vinylpyridineMe+_MeS-_m062x631+dp_h2o_TS.log	-803.4593334	-803.256896	-803.245252	-803.244308	-803.296325	109.479
2ethynylpyridineMe+_Cysteamine_m062x631+dp_h2o_QST2_TS_cont2.log	-897.3030223	-897.063135	-897.049049	-897.048105	-897.106938	123.825
5cyanopyrimidineMe+Cysteamine_m062x631+dp_h2o_TS_2.log	-929.4175888	-929.199603	-929.186191	-929.185246	-929.241014	117.373
2chloropyridine_m062x631+dp_h2o.log	-707.8	-707.7	-707.7	-707.7	-707.7	75.7
2chloropyridine_MeS-_m062x631+dp_h2o_scan_b_TSb.log	-1145.9	-1145.8	-1145.8	-1145.8	-1145.8	96.5
2chloropyridineMe+_m062x631+dp_h2o.log	-747.5	-747.4	-747.4	-747.4	-747.4	82.3
2chloropyridineMe+_Cl-_MeS-_m062x631+dp_h2o_scan_TS.log	-1646.0	-1645.8	-1645.8	-1645.8	-1645.9	119.8
3chloropyridine_m062x631+dp_h2o.log	-707.8	-707.7	-707.7	-707.7	-707.7	75.8
3chloropyridine_MeS-_m062x631+dp_h2o_scan_TS.log	-1145.9	-1145.8	-1145.8	-1145.8	-1145.8	97.4
3chloropyridineMe+_m062x631+dp_h2o.log	-747.5	-747.4	-747.4	-747.4	-747.4	85.0
3chloropyridineMe+_MeS-_m062x631+dp_h2o_scan_b_TSb_f.log	-1185.6	-1185.5	-1185.5	-1185.5	-1185.5	104.4
4chloropyridine_m062x631+dp_h2o.log	-707.8	-707.7	-707.7	-707.7	-707.7	75.7

4chloropyridine_MeS-_m062x631+dp_h2o_scan_TSb.log	-1145.9	-1145.8	-1145.8	-1145.8	-1145.8	92.3
4chloropyridineMe+_m062x631+dp_h2o.log	-747.5	-747.4	-747.4	-747.4	-747.4	84.5
4chloropyridineMe+_Cl-_MeS-_m062x631+dp_h2o_scan_TS.log	-1646.0	-1645.8	-1645.8	-1645.8	-1645.9	121.9
2chloropyrazine_m062x631+dp_h2o.log	-723.792	-723.724	-723.719	-723.718	-723.754	75.34
2chloropyrazine_MeS-_m062x631+dp_h2o_scan_TSb.log	-1161.94	-1161.84	-1161.83	-1161.83	-1161.87	92.21
3chloropyrazineMe+_m062x631+dp_h2o.log	-763.506	-763.396	-763.389	-763.388	-763.428	84.43
3chloropyrazineMe+_MeS-_m062x631+dp_h2o_scan_TS.log	-1201.69	-1201.55	-1201.54	-1201.54	-1201.58	100.9
2chloropyrimidine_m062x631+dp_h2o.log	-723.801	-723.733	-723.728	-723.727	-723.763	75.26
2chloropyrimidine_MeS-_m062x631+dp_h2o_scan_TS.log	-1161.96	-1161.85	-1161.84	-1161.84	-1161.89	94
2chloropyrimidineMe+_m062x631+dp_h2o.log	-763.512	-763.401	-763.395	-763.394	-763.433	81.88
2chloropyrimidineMe+_MeS-_m062x631+dp_h2o_scan_TS.log	-1201.69	-1201.55	-1201.54	-1201.54	-1201.59	107.3
5chloropyrimidine_m062x631+dp_h2o.log	-723.795	-723.726	-723.721	-723.72	-723.756	75.54
5chloropyrimidine_MeS-_m062x631+dp_h2o_scan_TS.log	-1161.94	-1161.83	-1161.82	-1161.82	-1161.87	97.18
5chloropyrimidineMe+_m062x631+dp_h2o.log	-763.51	-763.4	-763.393	-763.392	-763.433	84.7
5chloropyrimidineMe+_MeS-_m062x631+dp_h2o_scan_TS.log	-1201.69	-1201.54	-1201.53	-1201.53	-1201.58	100.1
6chloropyrimidine_m062x631+dp_h2o.log	-723.801	-723.732	-723.727	-723.726	-723.762	75.32
6chloropyrimidine_MeS-_m062x631+dp_h2o_scan_TSb.log	-1161.96	-1161.85	-1161.84	-1161.84	-1161.89	94.81
6chloropyrimidineMe+_m062x631+dp_h2o.log	-763.511	-763.4	-763.394	-763.393	-763.432	82
6chloropyrimidineMe+_Cl-_MeS-_m062x631+dp_h2o_scan_TS.log	-1662.05	-1661.9	-1661.88	-1661.88	-1661.94	120.2
